# A De Novo Arg649Trp Variant in GluA2 Impairs Its Assembly With GluA1: Associated With Epilepsy and Neurodevelopmental Disorders

**DOI:** 10.1155/humu/3227151

**Published:** 2026-10-01

**Authors:** Jing-Wen Chen, Ying-Chun Lin, Ping Hu, Feng-Chang Qiao, Yan Wang, Qing-Qing Li, Shi-Ji Deng, Qin-Xin Zhang, Jiang Chen, Yun Stone Shi

**Affiliations:** ^1^ Ministry of Education Key Laboratory of Model Animal for Disease Study Model Animal Research Center Medical School Nanjing University Nanjing China nju.edu.cn; ^2^ Department of Prenatal Diagnosis Women′s Hospital of Nanjing Medical University Nanjing Women and Children′s Healthcare Hospital Nanjing China; ^3^ Key Laboratory of Craniocerebral Diseases of Ningxia Hui Autonomous Region School of Basic Medical Sciences Ningxia Medical University Yinchuan China nxmu.edu.cn; ^4^ Department of Neurology Nanjing Drum Tower Hospital Affiliated Hospital of Medical School Nanjing University Nanjing China nju.edu.cn; ^5^ Guangdong Institute of Intelligence Science and Technology Zhuhai Hengqin China

## Abstract

AMPA‐type glutamate receptors (AMPARs) are the main mediators for fast excitatory synaptic transmission in the brain. Among AMPAR subunits, GluA2 is unique because of its role in forming Ca^2+^‐impermeable heteromeric receptors with GluA1/A3/A4, a property dictated by an arginine residue (GluA2(R)) in the pore region. In contrast, GluA2(R)‐lacking AMPARs are Ca^2+^‐permeable. Through whole‐exome sequencing (WES), we identified a de novo *GRIA2* variant (c.1945A>T, p.Arg649Trp) in a patient with neurodevelopmental disorders (NDDs) and epilepsy. Heterologous expression in HEK293T cells revealed that homomeric GluA2(Q)_Arg649Trp and heteromeric GluA1/A2(R)_Arg649Trp receptors retained near‐normal gating kinetics compared to wild‐type (WT). However, rectification index (RI) analysis demonstrated impaired assembly of GluA2(R)_Arg649Trp with GluA1. At 1:1 or 1:2 expression ratio (GluA1:GluA2), the Arg649Trp mutant exhibited a lower RI than WT, accompanied by reduced surface expression when coexpressed with GluA1. These results suggest that the Arg649Trp variant compromises the ability of GluA2 to incorporate into heteromeric AMPARs, leading to an increased proportion of GluA1 homomeric/Ca^2+^‐permeable receptors. Notably, recurrent missense variants at the M3–S2 linker of GluA2, including Ala639Ser and Thr646Asn, have also been independently identified to impair AMPAR assembly, suggesting this could be a common pathological mechanism. We thus propose that altered subunit stoichiometry, rather than changes in channel gating kinetics, represents a primary pathogenic mechanism for this *GRIA2* variant, contributing to NDD pathogenesis by elevating Ca^2+^‐permeable AMPARs in the brain.

## 1. Introduction

In the brain, alpha‐amino‐3‐hydroxy‐5‐methyl‐4‐isoxazolepropionic acid receptors (AMPARs) are ionotropic glutamate receptors that mediate fast excitatory synaptic transmission. Their subunit composition and synaptic localization critically regulate synaptic strength and plasticity, forming the molecular basis of learning and memory [1–4]. AMPARs are tetrameric complexes composed of four subunits (GluA1‐4), which assemble into homomeric or heteromeric channels [5]. Among these, GluA1/A2 heterotetramers represent the predominant subtype in the hippocampus and cortex [5]. Synaptic plasticity, including long‐term potentiation (LTP) and long‐term depression (LTD), has been shown to be fundamental to cognitive processes [6], and AMPARs play a central role in both the induction and maintenance of such plasticity [7, 8].

Among four AMPAR subunits, GluA2 is particularly critical in defining AMPAR properties. In the brain, nearly all GluA2 subunits undergo RNA editing at codon 607, converting a glutamine (Q) to an arginine (R) residue (Q/R editing) [9]. Unedited GluA2(Q) forms homomeric Ca^2+^‐permeable channels with inwardly rectifying current‐voltage (I‐V) relationship, whereas edited GluA2(R) alone does not generate functional channels because of extremely low conductance [10]. Instead, GluA2(R) preferentially assembles with GluA1, GluA3, or GluA4 to form heteromeric receptors that are Ca^2+^‐impermeable and exhibit linear I‐V relationship [11, 12]. Thus, GluA2 serves as a key determinant of AMPAR Ca^2+^ permeability and voltage‐dependent gating.

During early development, GluA2‐lacking Ca^2+^‐permeable AMPARs play essential roles in synaptogenesis and neuronal circuit formation. Ca^2+^ influx through these receptors promotes synapse maturation and functional connectivity in hippocampal neurons [13]. However, although transient Ca^2+^ permeability is necessary for neurodevelopment, its dysregulation can be detrimental: inhibition of Ca^2+^‐permeable AMPARs impairs axon growth in neural progenitor cells, whereas excessive Ca^2+^ influx induces excitotoxicity [14]. Thus, precise control of Ca^2+^‐permeable AMPAR expression is crucial for neuronal function. Notably, during maturation, GluA2‐containing Ca^2+^‐impermeable receptors gradually replace Ca^2+^‐permeable AMPARs, becoming the dominant synaptic population [15]. Disruption of this developmental switch leads to elevated GluA1:GluA2 ratio, prolonged Ca^2+^ permeability and neuronal excitotoxicity, a mechanism implicated in synaptic degeneration and neurological disorders [16].

To date, many neurodevelopmental disorders (NDD)‐related AMPAR variants have been reported. In general, NDDs, particularly epilepsy, are frequently linked to gain‐of‐function (GoF) variants in GluA subunits, which typically exhibit higher agonist affinity, increased surface expression, or prolonged deactivation/desensitization kinetics [17, 18]. Given GluA2 undergoes Q/R editing and is essential for forming Ca^2+^‐impermeable heteromeric AMPARs in most excitatory synapses, evaluating receptor assembly efficiency and subunit stoichiometry is thus critical for understanding the pathogenic mechanisms of GluA2 variants. An interesting pathogenic scenario unique to GluA2 is the loss‐of‐function (LoF) paradox. Even when a GluA2 variant acts as an LoF variant by disrupting its assembly with GluA1 or GluA3, it may paradoxically increase the abundance of GluA2‐lacking, Ca^2+^‐permeable AMPARs at the postsynaptic membrane. This aberrant Ca^2+^ permeability can trigger intracellular Ca^2+^ overload, ultimately leading to hyperexcitability of neuronal circuits and neurological dysfunction, a mechanism fundamentally distinct from conventional GoF or LoF in channel gating.

In this study, we identified a de novo *GRIA2* variant (c.1945A>T, p.Arg649Trp) in a patient with NDDs and epilepsy. When this variant was introduced in human cDNA of GluA2 and expressed in HEK cells, both homomeric GluA2(Q)_Arg649Trp and heteromeric GluA1/A2(R)_Arg649Trp receptors showed nearly normal gating kinetics compared with wild‐type (WT). However, I–V curve analysis demonstrated increased rectification for GluA2(R)_Arg649Trp when coexpressed with GluA1, suggesting impaired assembly into heteromeric complexes. Consistent with this finding, GluA2(R)_Arg649Trp exhibited reduced surface expression upon GluA1 coexpression. These data support a model in which the Arg649Trp variant disrupts GluA2 incorporation into heteromeric AMPARs, leading to increased Ca^2+^‐permeable, GluA2‐lacking AMPARs in the brain, a potential mechanism underlying the patient′s neurodevelopmental pathology. This finding likely fills a knowledge gap in the field that severe neurodevelopmental phenotypes can manifest in the absence of major kinetic defects in AMPAR function. The assembly defect may profoundly influence synaptic signaling and network excitability, in line with previous studies linking molecular alterations at the receptor level to circuit‐level dysfunction [19, 20].

## 2. Materials and Methods

### 2.1. Ethics Statement

The study was reviewed and approved by the Medical Ethics Committee of Nanjing Women and Children′s Healthcare Hospital, China (No. [2019] KY‐081). An informed consent was obtained from the patient′s parents prior to enrollment in this study and for publication of the related clinical data. All procedures performed in this study were in accordance with the ethical standards of the institutional research committee and with the Declaration of Helsinki and its later amendments.

### 2.2. Patient, Whole‐Exome Sequencing, and Variant Identification

The proband is a 5‐year‐old Chinese boy diagnosed with drug‐resistant epilepsy, intellectual disability, and language delay. He was born at term following an uncomplicated pregnancy and normal delivery. At current age, his growth parameters (height: 112 cm, weight: 20.51 kg, and occipitofrontal circumference [OFC]: 49 cm) fall within the 25th–50th percentile for his age. He developed onset of seizures at 3 months of age. Seizures manifested as focal myoclonic events, characterized by brief rhythmic clonic twitching of the face and unilateral upper limb, with preserved consciousness during attacks. At initial onset, seizures occurred 3–5 times per day, each lasting 10–15 s. EEG showed prominent spike and slow spikes over the right centrotemporal region. No ictal EEG patterns were captured during routine monitoring. According to the 2025 International League Against Epilepsy (ILAE) updated classification of epileptic seizures, the patient′s seizures were classified as focal preserved consciousness seizures.

Several antiepileptic treatments, including clonazepam (0.2 mg/kg/day for 6 months), topiramate (8 mg/kg/day for 5 months) and phenobarbital (5 mg/kg/day for 4 months), failed to control his epilepsy. He can now only say a few short intelligible phrases. His brain magnetic resonance imaging (MRI) and chromosomal microarray assay (CMA) revealed no apparent abnormalities.

Peripheral venous blood (2 mL) was collected from the proband and his parents using EDTA anticoagulation. Genomic DNA was extracted from the patient and their parents following the standard operating procedure of the QIAamp DNA extraction kit (QIAGEN, Hilden, Germany). The concentration and quality of the DNA were measured using a NanoDrop spectrophotometer (ThermoFisher Scientific, Waltham, Massachusetts, United States). Samples with a DNA quantity > 250 ng, DNA concentration > 30 ng/*μ*L, and an A260/280 ratio between 1.8 and 2.0 were selected for subsequent testing. The genomic DNA was fragmented by ultrasonication and then purified, followed by PCR amplification for library construction. The constructed libraries were subjected to quality control based on library concentration, effective molecular concentration, and DNA fragment size distribution. Targeted enrichment was performed according to the Agilent SureSelect QXT All Human Exon V6 kit (Agilent, Santa Clara, California, United States) instruction. The captured DNA underwent solid‐phase bridge amplification and was then sequenced on the Illumina NovaSeq 6000 platform (Illumina, Hayward, California, United States). The sequencing libraries were amplified into clusters via bridge PCR. Because of the reversible blocking of the 3′ end of dNTPs, the polymerase could only incorporate one base per cycle on the newly extended DNA strand. Fluorescence signals were collected under excitation light of a specific wavelength, allowing the identification of one base on the template strand in each cycle. The sequencing data underwent quality control, and the results were aligned to the human reference genome sequence (GRCh37/hg19) using BWA0.6.2‐r126 software (https://sourceforge.net/projects/bio-bwa/). Redundant information generated during library construction, such as adapter sequences and repetitive sequences, was removed. GATK software (http://www.broadinstitute.org/gatk/) was used for realignment and base quality recalibration to identify single nucleotide variants (SNVs) and small insertions or deletions in the genome. The identified variants were annotated and classified using the ANNOVAR annotation tool (https://annovar.openbioinformatics.org/en/latest/). Polymerase chain reaction (PCR) was performed to amplify the affected fragment of *GRIA2* using specific primers (forward primer: 5 ^′^‐TCTGGTTTTCCTTGGGTG‐3 ^′^ and reverse primer: 5 ^′^‐CAGAGGGCTCCGCACTCC‐3 ^′^), and the purified PCR products were applied to Sanger sequencing to confirm the variant. Trio‐based WES of the proband and his parents yielded approximately 72.78 million 150‐bp reads; 99.65% of reads were aligned to the human reference genome, with 3988.36 Mb mapped to the target region at a mean coverage of 100.52×. A total of 57,960 single‐nucleotide polymorphism (SNP) sites were identified, including 47,457 nonsynonymous SNPs in coding sequences and 1,847 in splice sites, together with 6355 indels (565 in coding sequences and 580 in splice sites). Candidate variants were prioritized using a stepwise filtration strategy based on allele frequency, inheritance pattern, variant type and predicted pathogenicity.

### 2.3. Plasmid Construction

Human GluA1, as well as GluA2(Q) and GluA2(R) cDNAs were subcloned into expression vectors, namely pCAGGS‐hGluA1‐IRES‐GFP, pCAGGS‐HA‐hGluA2(Q)‐IRES‐mCherry and pCAGGS‐HA‐hGluA2(R)‐IRES‐mCherry. An HA‐epitope was inserted after the signal sequence of GluA2 to assist in the examination of GluA2 surface expression. Overlapping PCR was used to introduce the c.1945A>T mutation into GluA2(Q) and GluA2(R) coding DNA sequences (CDSs). The mutations were verified using Sanger sequencing.

### 2.4. Cell Culture and Transfection

HEK293T cells were provided from Procell (Wuhan, China). The cell line was authenticated by STR profiling with correct identity and confirmed to be free of mycoplasma contamination prior to experimentation. HEK293T cells were cultured in high‐glucose DMEM medium (Gibco, Grand Island, New York, United States) supplemented with 10% fetal bovine serum (FBS, Gibco) at 37°C and 5% CO₂ conditions. When the cell confluence reached 50% –60%, the plasmid vectors were transfected into the cells using Lipofectamine 2000 reagent (ThermoFisher Scientific). NBQX (100 *μ*M, Alomone Labs, Jerusalem, Israel) was included in the culture medium after transfection to prohibit the cytotoxicity of AMPARs.

### 2.5. Electrophysiology

The electrophysiological recordings were processed as [21]. Briefly, 24 h after transfection, the HEK293T cells were trypsinized (with 0.25% trypsin‐EDTA, Gibco) and then replated on poly D‐lysine (Sigma‐Aldrich, Saint Louis, Missouri, United States) coated glass slices. After overnight culture, the cells were used for patch‐clamp recording. The cells were bathed in the extracellular solution containing (in mM) NaCl 140, KCl 2.5, CaCl_2_ 2, MgCl_2_ 1, HEPES 10 and D‐Glucose 5, adjusted to pH 7.4 and 290–300 mOsm. 3–5 M*Ω* borosilicate glass recording electrode was filled with intracellular solution containing (in mM) KF 130, KOH 33, MgCl_2_ 2, CaCl_2_ 1, EGTA 11, HEPES 10 and spermine 0.1, adjusted to pH 7.4 and 290–300 mOsm. All the ingredients in the extracellular and intracellular solution were from Sigma‐Aldrich. For the recordings of deactivation and desensitization, the outside‐out configuration was established. For the recordings of I–V relationship, whole‐cell configuration was established. The fast administration of glutamate relied on a *θ*‐shape glass pipette, with one tube filled with basic extracellular solution and the other filled with extracellular solution containing 10 mM glutamate (Sigma‐Aldrich), mounted on a piezoelectric bimorph, and the liquid flow was driven by gravity. Glutamate‐mediated currents for the analysis of deactivation and desensitization were recorded at −70 mV holding voltage. Glutamate‐mediated currents for the analysis of I–V curve and rectification index (RI) were recorded at the range of −100 mV to +100 mV with 20 mV interval. The data were collected with Axon Digidata 1550 system and Multiclamp 700B amplifier (Molecular Devices, San Jose, California, United States), digitized at 50 kHz, and filtered at 2 kHz. For heteromeric GluA1/GluA2(R) expression, plasmids encoding GluA1 and GluA2(R) were cotransfected at mass ratios of 1:1, 1:2, or 1:4 (GluA1:GluA2).

### 2.6. Immunofluorescent Staining

After 24 h of transfection, the HEK293T cells were washed twice with 0.01 M PBS (in mM, NaCl 137, KCl 2.7, Na_2_HPO_4_ 10, KH_2_PO_4_ 1.8) for 5 min each. The cells were fixed with 4% paraformaldehyde (PFA, Biotend, Shanghai, China) at 4°C for 10 min, followed by three 10‐min washes with 0.01 M PBS. The coverslips were then blocked with goat serum (Biosharp, Beijing, China) at room temperature for 30 min. After removing the blocking solution, the cells were incubated with mouse‐hosted anti‐HA primary antibody (H3663, Sigma‐Aldrich; diluted 1:500 in ready‐to‐use serum, BL1097A, Biosharp) at 4°C overnight. The coverslips were washed three times with 0.01 M PBS for 10 min each. Alexa Fluor 488‐conjugated donkey‐host antimouse IgG secondary antibody (BS10015, Bioworld, Bloomington, Minnesota, United States; diluted 1:500 in 0.01 M PBS) was added and incubated at room temperature for 1 hour, followed by washing with 0.01 M PBS to complete the staining of membrane‐bound GluA2(Q) and GluA2(R). Subsequently, the cells were further blocked with goat serum containing 0.3% Triton X‐100 (Beyotime, Shanghai, China) at room temperature for 30 min to permeabilize the cell membrane. Intracellular GluA2(Q) and GluA2(R) were stained with rabbit‐host anti‐HA primary antibody (3724, Cell Signaling Technology, Danvers, Massachusetts, United States; diluted 1:500 in ready‐to‐use serum) at 4°C overnight. The coverslips were washed three times with 0.01 M PBS for 10 min each, followed by incubation with Alexa Fluor 594‐conjugated donkey‐hosted antirabbit IgG secondary antibody (BS10030, Bioworld; diluted 1:500 in 0.01 M PBS) at room temperature for 1 h. After washing three times with 0.01 M PBS for 10 min each, the coverslips were mounted onto glass slides using the DAPI (BS097, Biosharp)‐containing mounting medium diluted 1:1000 in 0.01 M PBS. Fluorescent images were observed and captured using a confocal laser‐scanning microscope (LSM880, ZEISS, Oberkochen, Germany). Appropriate excitation and emission wavelengths were selected for different fluorophores, Alexa Fluor 488 (488‐nm excitation wavelength and 520‐nm emission wavelength) and Alexa Fluor 594 (594‐nm excitation wavelength and 619‐nm emission wavelength). Finally, ImageJ software (ij154, Bethesda, Maryland, Unite States) was used to analyze the positive results of the immunoreactivity.

## 3. Statistics

The data were presented as mean ± SEM. The comparisons between two groups utilized unpaired Student′s *t*‐test. The comparisons of I–V curves utilized two‐way ANOVA with Bonferroni′s multiple comparison test. *p* < 0.05 was considered statistically significant.

## 4. Results

### 4.1. Identification of a De Novo GluA2_Arg649Trp Variant

Analyses of whole‐exome sequences from the proband and his parents identified a heterozygous missense variant in exon 12 of the *GRIA2* gene in the q32.1 region of chromosome 4 (NM_000826.6: c.1945A>T, p.Arg649Trp), which was absent in both parents, indicating a de novo occurrence (PS2_Moderate; Figure 1a). The heterozygous A‐to‐T substitution at nucleotide position 1945 results in replacement of the positively charged arginine (Arg) at position 649 of GluA2 by a hydrophobic tryptophan (Trp). The variant is located in the linker between the third transmembrane domain (M3) and the second segment of the ligand‐binding domain (S2) of GluA2 (PM1; Figure 1b), where several other variants have been reported previously [18]. The variant is absent from the ExAC, dbSNP, 1000 Genomes and gnomAD (v2.1.1 and v3.1.2) databases, and no homozygous or compound heterozygous carriers have been reported (PM2_Supporting). Missense variants of *GRIA2* are a major cause of NDDs with language impairment and behavioral abnormalities (MIM 618917; PP2). Multiple in silico prediction tools (SIFT, Polyphen2_HVAR, PROVEAN, MutationTaster, M‐CAP, REVEL, GERP, phyloP20way, and phastCons20way) predict that this variant is damaging to protein function (PP3). Pathogenicity classification followed the 2015 ACMG/AMP guidelines [22], with evidence‐strength adjustments implemented according to ClinGen‐SVI consensus recommendations: PS2 was downgraded from strong to moderate because the clinical phenotype was not highly gene‐specific; PM2 was downgraded from moderate to supporting in accordance with the SVI recommendation for the PM2 criterion. Combining the above evidence (PS2_Moderate, PM1, PM2_Supporting, PP2 and PP3), the GluA2_Arg649Trp variant was classified as likely pathogenic [23]. It is worth noting that the Arg649 residue is highly conserved across vertebrate species (Figure 1c), implying a conserved function of this site in GluA2.

**Figure 1:**
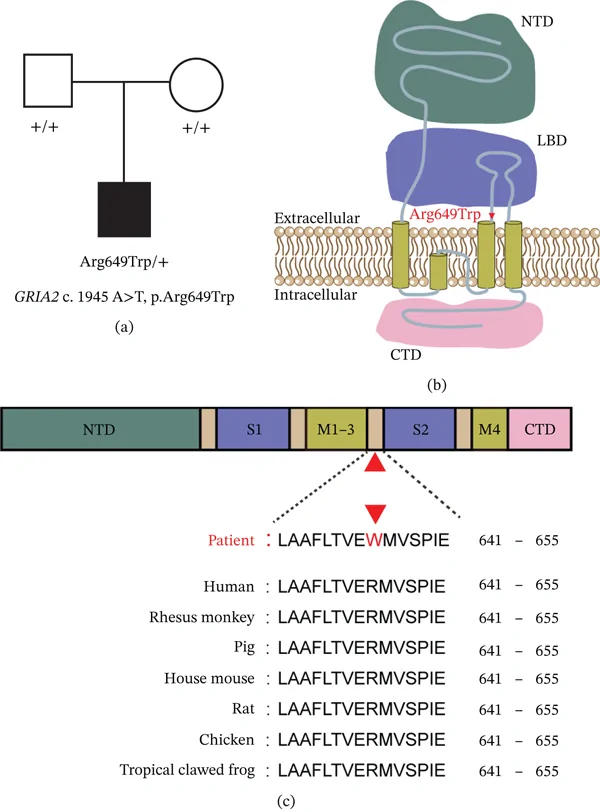
Pedigree and identification of the GluA2_Arg649Trp variant. (a) Family pedigree of the rare variant. (b) Topology model of GluA2. NTD, N‐terminal domain; LBD, ligand‐binding domain; CTD, C‐terminal domain; red triangle, location of Arg649Trp. (c) Sequence alignment of GluA2 around Arg 649 among species.

### 4.2. Gating Kinetics of GluA2_Arg649Trp

Deactivation and desensitization represent two primary gating kinetic properties reflecting receptor decay [21]. Here, we heterogeneously expressed GluA2 and Arg649Trp variants in HEK293T cells and employed a *θ*‐shape borosilicate glass tube attached to a piezoelectric bimorph to achieve the submillisecond scale fast delivery of glutamate. The GluA2(Q)_Arg649Trp receptor exhibited slightly slower deactivation kinetics than WT GluA2(Q) (Figure 2a), with no difference in desensitization (Figure 2b). In GluA1/GluA2(R) heteromeric receptors, the Arg649Trp variant did not affect deactivation (Figure 2c), but slightly reduced the desensitization time (Figure 2d). These results indicate that the Arg649Trp variant minimally affects the deactivation and desensitization of both the GluA2(Q) homomeric receptor and the GluA1/GluA2(R) heteromeric receptor, suggesting that haploinsufficiency, defined as a ~ 50% reduction in functional protein, is unlikely to account for the pathogenic effect.

**Figure 2:**
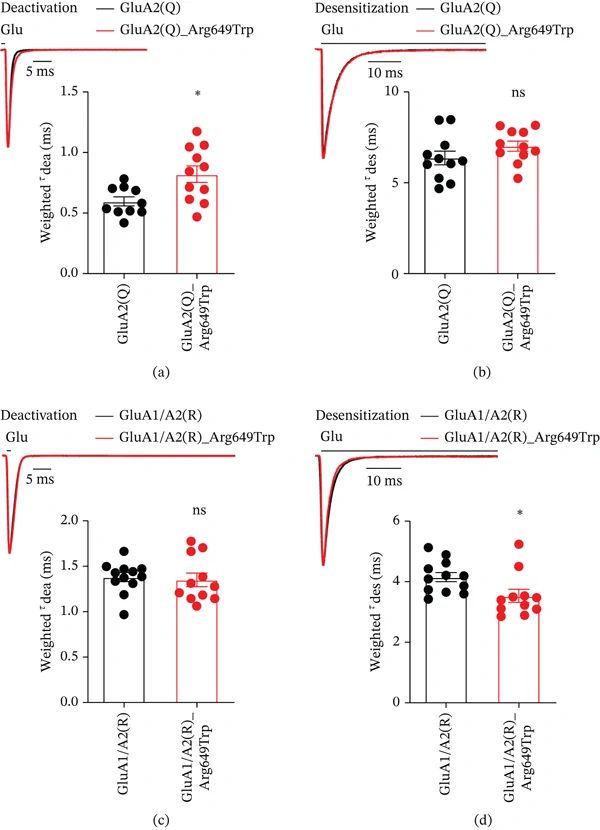
Arg649Trp variant modestly affected deactivation and desensitization of GluA2. (a,b) Decay kinetics of WT and Arg649Trp‐mutant GluA2(Q) homomeric receptors. (a) Deactivation, GluA2(Q), *τ* = 0.60 ± 0.04 ms, *n* = 10; GluA2(Q)_Arg649Trp, *τ* = 0.82 ± 0.07 ms, *n* = 11,  ^∗^ *p* = 0.0112. (b) Desensitization, GluA2(Q), *τ* = 6.36 ± 0.38 ms, *n* = 11; GluA2(Q)_Arg649Trp, *τ* = 7.02 ± 0.28 ms, *n* = 11, ns, *p* = 0.6109. (c,d) Decay kinetics of GluA1/A2(R) heteromeric receptors with 1:1 GluA1‐to‐GluA2 mass ratio. (c) Deactivation, GluA1/A2(R), *τ* = 1.38 ± 0.05 ms, *n* = 12; GluA1/A2(R)_Arg649Trp, *τ* = 1.35 ± 0.08 ms, *n* = 11, ns, *p* = 0.7619. (d) Desensitization, GluA1/A2(R), *τ* = 4.14 ± 0.15 ms, *n* = 12; GluA1/A2(R)_Arg649Trp, *τ* = 3.52 ± 0.22 ms, *n* = 11,  ^∗^ *p* = 0.0278. Unpaired *t*‐tests in a–d. Data are presented as mean ± SEM.

### 4.3. Rectification Properties of GluA1/GluA2_Arg649Trp

The GluA1/A2(R) heteromeric receptor typically exhibits a linear I–V relationship, in contrast to homomeric AMPARs, which display inward rectification because of voltage‐dependent polyamine blockade [11]. At 1:1 and 1:2 GluA1:GluA2 mass ratios, the Arg649Trp variant displayed stronger inward rectification than WT (Figure 3a,b,d,e), with a significantly reduced RI (Figure 3c,f). Increasing the proportion of GluA2 (WT or Arg649Trp) shifted the I–V curves toward linearity at all tested ratios (Figure 3d,e,g,h), and the difference between WT and Arg649Trp disappeared at the 1:4 ratio (Figure 3g–i). These findings indicate that at lower GluA2 expression levels (1:1 and 1:2), the Arg649Trp variant significantly increases the proportion of GluA1 homomeric receptors at the cell surface.

**Figure 3:**
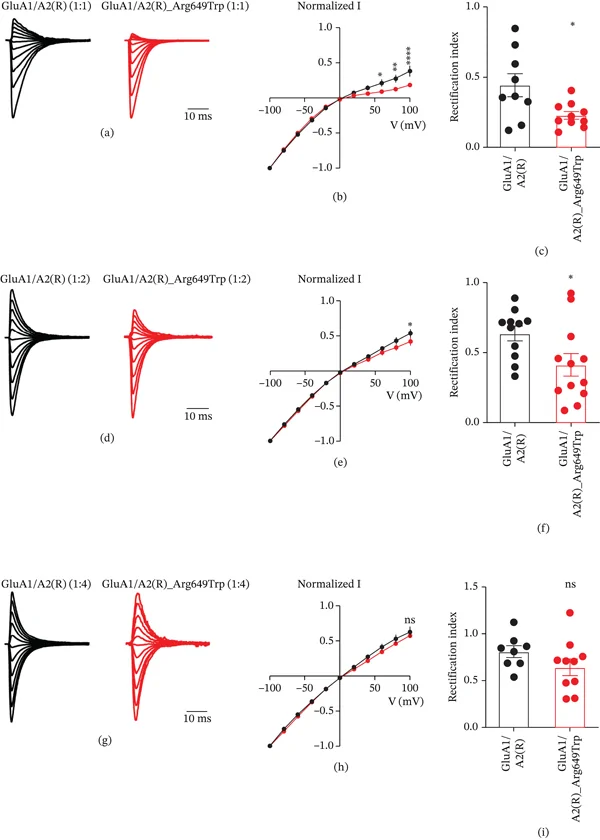
GluA2(R)_Arg649Trp variant enhanced rectification. (a–c) I–V relationships of GluA1/A2(R) and GluA1/A2(R)_Arg649Trp with 1:1 mass ratio, GluA1/A2(R), *n* = 9; GluA1/A2(R)_Arg649Trp, *n* = 10. (a) Sample traces of 500‐ms glutamate‐induced currents from −100 mV to + 100 mV with 20‐mV interval. (b) I–V curves,  ^∗^ *p* = 0.0142 at +60 mV,  ^∗∗^ *p* = 0.0013 at +80 mV,  ^∗∗∗∗^ *p* < 0.0001 at +100 mV; ns, *p* > 0.05, at other holding potentials. (c) Bar graph of RI, WT, 0.44 ± 0.08; Arg649Trp, 0.23 ± 0.03,  ^∗^ *p* = 0.0192. (d–f) I–V relationships of GluA1/A2(R) and GluA1/A2(R)_Arg649Trp with 1:2 mass ratio, GluA1/A2(R), *n* = 11; GluA1/A2(R)_Arg649Trp, *n* = 12. (d) Sample traces. (e) I–V curves,  ^∗^ *p* = 0.0414 at +100 mV; ns, *p* > 0.05, at other holding potentials. (f) Bar graph of RI, WT, 0.64 ± 0.05; Arg649Trp, 0.41 ± 0.08,  ^∗^ *p* = 0.0321. (g–i) I–V relationships of GluA1/A2(R) and GluA1/A2(R)_Arg649Trp with 1:4 mass ratio, GluA1/A2(R), *n* = 8; GluA1/A2(R)_Arg649Trp, *n* = 10. (g) Sample traces. (h) I–V curves, ns, *p* > 0.05, at all holding potentials. (i) Bar graph of RI, WT, 0.81 ± 0.06; Arg649Trp, 0.66 ± 0.09, ns, *p* = 0.1595. Unpaired *t*‐tests in c, f, and i. Two‐way ANOVA with Bonferroni′s multiple comparison test in b, e, and h. Data are presented as mean ± SEM.

### 4.4. GluA2_Arg649Trp Reduces the Surface Expression of GluA1/A2 Heteromeric Receptors

We performed immunofluorescent staining to investigate the effect of Arg649Trp variant on receptor surface expression. A hemagglutinin (HA) tag was inserted immediately downstream of the signal peptide of GluA2 constructs. The cell‐surface GluA2 was labeled with a mouse anti‐HA antibody before membrane permeabilization, and the intracellular GluA2 with a rabbit anti‐HA antibody after permeabilization. The surface‐to‐intracellular ratio of HA fluorescence was comparable between GluA2(Q) and GluA2(Q)_Arg649Trp (Figure 4a,b), but was significantly lower for GluA1/GluA2(R)_Arg649Trp than for the WT control (Figure 4c,d). These results demonstrate that the Arg649Trp variant specifically reduces the surface expression of GluA1/GluA2 heteromeric receptors, indicating that it impairs GluA1/GluA2 heteromeric assembly.

**Figure 4:**
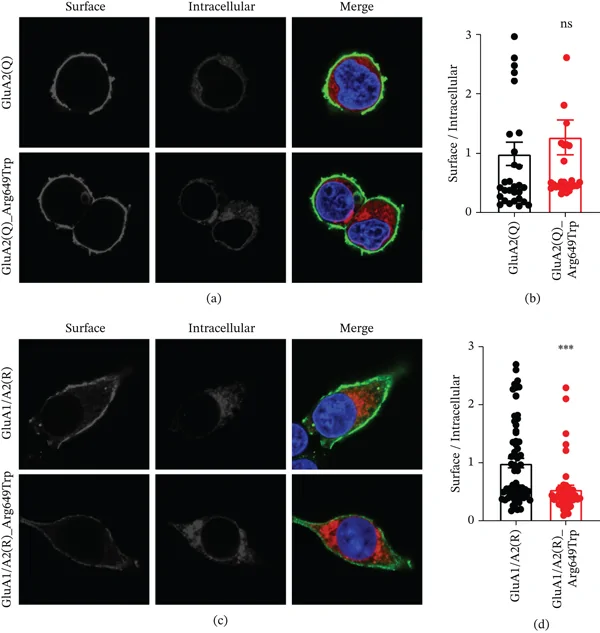
Immunostaining of HA‐GluA2 in HEK293T cells coexpressing GluA1 and GluA2 variants. (a,b) Overexpression of HA‐tagged GluA2(Q) and GluA2(Q)_Arg649Trp in HEK293T cells. (a) Representative images of immunofluorescence staining. Alexa Fluor 488 (green) labeled on the surface GluA2, Alexa Fluor 594 (red) labeled the intracellular GluA2, and DAPI (blue) labeled on the nucleus, scale bar 10 *μ*m. (b) Plot of statistical results for the normalized ratio of membrane to intracellular expression of GluA2(Q). GluA2(Q), *n* = 30; GluA2(Q)_Arg649Trp, *n* = 30, ns, *p* = 0.4410. (c,d) Overexpression of HA‐tagged GluA1/A2(R) (1:1) and GluA1/A2(R)_Arg649Trp (1:1) in HEK293T cells. (c) Representative images of immunofluorescence staining of GluA2(R). Label colors and scale bar are the same as those in a. (d) plot of statistical results for the normalized ratio of membrane protein to intracellular protein expression of GluA2(R), GluA1/A2(R) (1:1), *n* = 65; GluA1/A2(R)_Arg649Trp (1:1), *n* = 43,  ^∗∗∗^ *p* = 0.0005. Unpaired *t*‐tests in b and d. Data are presented as mean ± SEM.

## 5. Discussion

The central implication of this study is that a *GRIA2* variant can cause neurodevelopmental pathology primarily by altering subunit assembly and stoichiometry, rather than by disrupting channel gating. We identified a de novo GluA2_Arg649Trp variant in a patient with drug‐resistant epilepsy and intellectual disability; functional characterization revealed only minor effects on AMPAR gating. Instead, our data demonstrate that this variant impairs the heteromeric assembly of GluA2 with GluA1, reducing the surface expression of GluA2‐containing receptors and increasing the proportion of Ca^2+^‐permeable GluA1 homomeric AMPARs. These findings support a pathogenic model in which disruption of normal GluA1/GluA2 stoichiometry, and the resulting alteration in Ca^2+^ permeability, contribute to NDDs even in the absence of major gating defects.

Arg649 lies in the linker between the M3 and S2 domains, a region extensively studied for its role in channel gating. Substitution of this residue by a negatively charged glutamate in rodent GluA2 (Arg649Glu), or at the homologous positions in GluA1 (Arg642Glu) or GluK2 (Arg663Glu), impairs channel desensitization [23]. In addition, we previously identified patients carrying variants at the homologous site in GluA3 (Arg660Thr/Ser) that significantly impaired channel desensitization [17, 21]. Therefore, the modest impact of Arg649Trp on the gating of GluA2‐containing receptors is unexpected, highlighting residue‐specific effects of variants at this site. Generally, the N‐terminal domain (NTD) is critical for heteromeric AMPAR assembly [24, 25]. Salpietro et al. reported multiple *GRIA2* variants in patients and found that Gly47Glu and Asp302Gly, located in the NTD, affect GluA1/A2 assembly [18]. Interestingly, the study also found that Ala639Ser and Thr646Asn, close to Arg649Trp reported here, impaired assembly with GluA1 and reduced surface expression. Together with the present case, these independent observations indicate that the M3–S2 linker not only participates in gating but also plays a critical role in AMPAR assembly, though the underlying mechanism remains to be understood.

Variants of *GRIA2* are associated with NDDs, including developmental delay, intellectual disability, language impairment, epileptic encephalopathy, and autism spectrum disorder [18, 26]. To date, more than 30 pathogenic or likely pathogenic GluA2 missense variants have been reported, covering most structural domains [18, 26–36] (Figure 5). For AMPARs, GoF variants are more frequently associated with epilepsy, a disease related to enhanced and uncontrolled activity of neural networks. In GluA2, Ala643Val shows markedly slowed deactivation and desensitization, a typical GoF phenotype, and the patient was effectively treated with the AMPAR antagonist perampanel [30]. In addition, Glu508Val, Val647Ala, and Val647Leu increase glutamate potency [36], which, though not directly examined, is mostly associated with slowed deactivation. Unlike the above variants, Arg649Trp showed little effect on gating kinetics; rather, it interrupts GluA1/A2 heteromeric assembly and enhances Ca^2+^ permeability of AMPARs. Such molecular aberrations may hypothetically induce neuronal excitotoxicity, impair synaptic plasticity, and ultimately remodel brain network dynamics [37, 38], triggering large‐scale abnormal brain activity and seizure [39, 40]. Reduced ability in heteromeric assembly represents a LoF of GluA2 subunit, but enhanced Ca^2+^ permeability is certainly a GoF of AMPARs. Thus Arg649Trp may represent an atypical GoF for *GRIA2* variants. In this regard, we predict that perampanel might be effective in patients with assembly‐defective *GRIA2* variants through inhibition of excessive Ca^2+^‐permeable AMPARs, which surely requires to be evaluated in future studies with larger cohorts.

**Figure 5:**
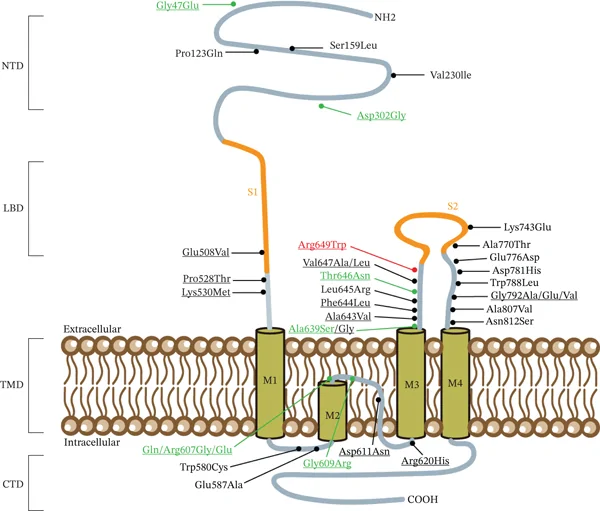
Schematic summary of likely pathogenic GluA2 missense variants. These variants, except for Arg649Trp, were identified in previous publications [18, 26–36]. Underlined variants have been functionally tested; green‐labeled variants affected the rectification of GluA1 and GluA2 coexpression. NTD, N‐terminal domain; LBD, ligand‐binding domain (including S1 and S2); TMD, transmembrane domain; CTD, C‐terminal domain.

Recently, Allen et al. [41] showed that *GRID1* and *GRID2* variants can act either by disrupting trans‐synaptic complex assembly or by conferring constitutive channel activity. Similarly, our findings extend the growing recognition that glutamate receptor variants cause NDDs through diverse, variant‐specific mechanisms beyond classical gain‐ or LoF in channel gating.

Several limitations of this study should be acknowledged. First, functional characterization was performed in HEK293T cells, which do not fully recapitulate the native synaptic environment of neurons, including auxiliary subunits, such as TARPs, that modulate AMPAR trafficking and gating. Second, we assessed surface expression by immunofluorescence rather than quantitative biochemical methods, so the precise change in receptor stoichiometry was not directly measured. Third, although we identified assembly defects caused by variants in the M3–S2 linker, the molecular mechanism by which this region regulates AMPAR assembly remains to be determined. More broadly, the pathophysiological relevance of the proposed assembly‐defect mechanism requires validation: (i) replication in additional independent patients carrying M3–S2 linker variants; (ii) confirmation in disease‐relevant cellular or animal models; and (iii) broader functional surveys across the recurrent linker variant class to establish the general applicability of this mechanism beyond Arg649Trp.

## 6. Conclusions

In summary, our study identifies impaired subunit assembly as a novel pathogenic mechanism for *GRIA2* variants. Together with a previous study [18], our findings demonstrate that impaired AMPAR subunit assembly can underlie NDD pathogenesis. Conceptually, this defective GluA1/A2 assembly causes dysregulated synaptic Ca^2+^ signaling, which induces circuit hyperexcitability and ultimately mediates the epilepsy and neurodevelopmental phenotypes (Figure 6). Our report expands the functional spectrum of pathogenic *GRIA2* variants and highlights the M3–S2 linker as a critical determinant of AMPAR assembly, suggesting calcium homeostasis disruption as a potential therapeutic target. Additional patients and structural studies linking M3–S2 variants to assembly defects would further advance our understanding in this hypothetical pathogenic mechanism.

**Figure 6:**
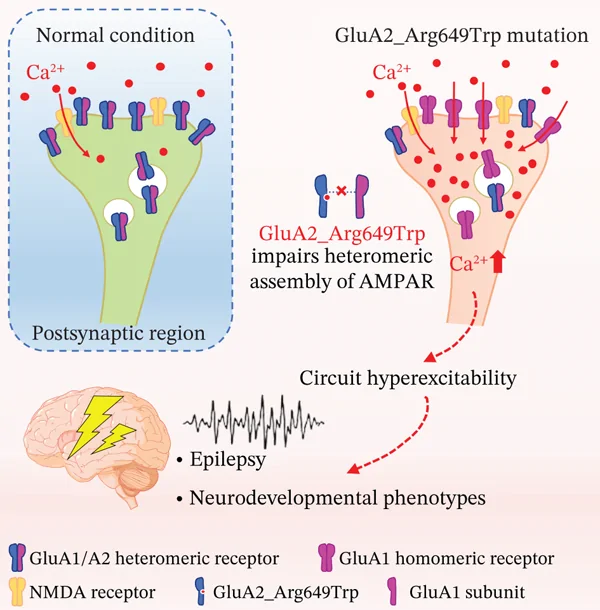
Schematic illustrating the pathophysiological mechanisms linking the GluA2_Arg649Trp variant to epilepsy and neurodevelopmental phenotypes. GluA2_Arg649Trp variant impairs GluA1/A2 assembly, reducing surface expression of Ca^2+^‐impermeable heteromeric receptors and relatively increasing surface Ca^2+^‐permeable GluA1 homomers. The resulting dysregulated synaptic Ca^2+^ signaling leads to circuit hyperexcitability, ultimately causing epilepsy and neurodevelopmental phenotypes.

## Author Contributions

Q.X.Z., J.C. and Y.S.S. designed the study. J.W.C., Y.C.L., Q.Q.L., and S.J.D. performed electrophysiological and biochemical experiments. F.C.Q., Y.W., and P.H. collected clinical information and performed the genetic analysis. Q.X.Z., J.C., and Y.S.S. wrote the manuscript. J.W.C. and Y.C.L., have contributed equally to this work.

## Funding

This study was supported by the National Natural Science Foundation of China, 10.13039/501100001809 (82271891; 32170951; 82101943; 82371862 823B2027; 82460232); the National Key Research and Development Program of China, 10.13039/501100012166, 2022YFC2703400; and the Fundamental Research Funds for the Central Universities, 10.13039/501100012226, 021414380533.

## Conflicts of Interest

The authors have nothing to report.

## Data Availability

The data that support the findings of this study are available on request from the corresponding author. The data are not publicly available due to privacy or ethical restrictions.
